# Advances and Challenges for QTL Analysis and GWAS in the Plant-Breeding of High-Yielding: A Focus on Rapeseed

**DOI:** 10.3390/biom11101516

**Published:** 2021-10-15

**Authors:** Shahid Ullah Khan, Sumbul Saeed, Muhammad Hafeez Ullah Khan, Chuchuan Fan, Sunny Ahmar, Osvin Arriagada, Raheel Shahzad, Ferdinando Branca, Freddy Mora-Poblete

**Affiliations:** 1 National Key Laboratory of Crop Genetic Improvement, Huazhong Agricultural University, Wuhan 430070, China; shahidbiochem@webmail.hzau.edu.cn (S.U.K.); sumbulsaeed@webmail.hzau.edu.cn (S.S.); hafeezbiotech@webmail.hzau.edu.cn (M.H.U.K.); 2 Institute of Biological Sciences, University of Talca, 1 Poniente 1141, Talca 3465548, Chile; sunnyahmar13@gmail.com; 3 Departamento de Ciencias Vegetales, Facultad de Agronomía e Ingeniería Forestal, Pontificia Universidad Católica de Chile, Santiago 7820436, Chile; arriagada.lagos.o@gmail.com; 4 Department of Biotechnology, Faculty of Science & Technology, Universitas Muhammadiyah Bandung, Bandung 40614, Indonesia; raheel.shahzad957@gmail.com; 5 Department of Agriculture, Food and Environment (Di3A), University of Catania, 95123 Catania, Italy; fbranca@unict.it

**Keywords:** quantitative trait loci, multi-locus GWAS, yield-related traits, high-throughput genotyping

## Abstract

Yield is one of the most important agronomic traits for the breeding of rapeseed (*Brassica napus* L), but its genetic dissection for the formation of high yield remains enigmatic, given the rapid population growth. In the present review, we review the discovery of major loci underlying important agronomic traits and the recent advancement in the selection of complex traits. Further, we discuss the benchmark summary of high-throughput techniques for the high-resolution genetic breeding of rapeseed. Biparental linkage analysis and association mapping have become powerful strategies to comprehend the genetic architecture of complex agronomic traits in crops. The generation of improved crop varieties, especially rapeseed, is greatly urged to enhance yield productivity. In this sense, the whole-genome sequencing of rapeseed has become achievable to clone and identify quantitative trait loci (QTLs). Moreover, the generation of high-throughput sequencing and genotyping techniques has significantly enhanced the precision of QTL mapping and genome-wide association study (GWAS) methodologies. Furthermore, this study demonstrates the first attempt to identify novel QTLs of yield-related traits, specifically focusing on ovule number per pod (ON). We also highlight the recent breakthrough concerning single-locus-GWAS (SL-GWAS) and multi-locus GWAS (ML-GWAS), which aim to enhance the potential and robust control of GWAS for improved complex traits.

## 1. Introduction

Rapeseed (*Brassica napus* L., genome AACC, 2n = 38) is one of the second top-most oilseed crops predominantly grown for protein meal and vegetable oil across the world [1,2,3,4]. Feeding the ever-expanding population is a major challenge due to the ever-expanding demand from humans and the production of biofuels increases global food security [5,6]. To fulfill the global food demand, grain production is expected to increase up to 50% by 2025 [7]. Therefore, to accomplish this exploiting demand, different plant varieties with improved agronomic traits will steadily need to be generated. Various agronomic traits include stress-inducive response, yield and yield-related traits, which are controlled by many genes that are being significantly influenced by the environment [8]. Therefore, to elaborate the actual mechanism of agronomic traits, the dissection and isolation of complex traits into the single chromosome locus and their characterization for each quantitative trait locus (QTL) is imperatively noticed.

With the rapid progress in sequencing technology and bioinformatics tools, QTL analysis, offering the ever-increasing opportunity, has been a highly significant, precise and efficient genotyping approach that utilizes molecular markers (e.g., single nucleotide polymorphism, SNPs) by engineering a powerful marker-based selection system that stringently controls the complex genomic traits [9]. For gene mapping, the population of 60 K illumines Infinium SNP arrays for *B. napus* can be sustainably transferred to a gene-based, low-cost and high-throughput genotype-based screening method [10]. To control the desired trait, this system is tremendously effective in mapping the QTLs within a narrow-range genomic level; it can also be the source of supply markers within the desired traits [11]. A previous study has incredibly triggered the rapid outcomes between *Arabidopsis* and *Brassica* species. These outcomes showed that 12 genes have been identified with 8 quantitative trait nucleotides (QTNs) underlying seed weight. Moreover, a single gene-specific marker (BnAP2) was also identified [12].

Linkage disequilibrium (LD) mapping, also known as association mapping, demonstrates the statistical relationship between the genetic markers and phenotypes within the natural populations, which constitutes an efficient approach for the association mapping of QTL traits. Genome-wide association studies (GWAS) are an effective and promising approach for partitioning complex traits [13,14]. More recently, GWAS has been promisingly involved for many crop varieties [15], including *Avena sativa* [16], *Sorghum bicolor* [17], *Hordeum vulgare* [18], *Triticum aestivum* [19], *Glycine max* [20], *Oryza sativa* [21], *Zea mays* [22], *Arachis hypogaea* [23] and *Brassica napus* [24].

The analysis of these crops by QTL/GWAS methods will be rapidly expanding and applied to cereals crops. Therefore, the present study mainly focuses on the rapeseed QTL characteristics that are an important model for future research. The current review also provides a benchmark summary of the recently studied literature with a major concern on rapeseed QTLs that demonstrate a significant role in future breeding strategies. Furthermore, this study also highlights the comprehensive information about both the single-locus GWAS (SL-GWAS) and multi-locus GWAS (ML-GWAS) approaches, which can expand the robustness of GWAS for complex genetic traits.

## 2. Breeding Objectives in Rapeseed Crop

To focus on breeding strategies in almost all cultivated plants to improve their yield, more specifically, seed yield is our major target. In rapeseed, seed yield remains an important breeding goal. In particular, oil quality, low erucic acid and glucosinolates contents have been important aspects for breeding in rapeseed cultivars [25,26]. These efforts have been utilized to reduce linolenic acid and to improve oleic acid contents, shelf-life and palatability of the oil for human consumption [27,28] and have led to the development of double-low cultivars [25,29], known as “HOLLi”. In addition to oil quality, seed oil contents are also becoming a key target. Researchers have concentrated their attention on exploring the genetic effects and mechanisms controlling oil production [30] to satisfy edible oil and biofuels production requirements. In this regard, several QTLs have been identified maintaining oil contents [31,32] and improving the quality of rapeseed breeding. The targets are not only focused on seed oil content but also on protein content to enhance the energy value of meals for livestock as a feed resource. To improve the feed meal, researchers have focused on reducing the level of glucosinolates, tannins and sinapate esters [26]. Genetic engineering strategies have successfully improved the seed protein content by increasing the utility of essential amino acids.

Further objectives have been focused on quality improvement, including reducing tannin contents in the seed protein and decreased fiber content [25,26] to boost meal quality and palatability; as mentioned, seed yield is the priority effort of breeders. According to Nesi et al. [26], seed yield has been increased by 50% during the last fifty years. However, the increasing demands on the plant’s edible oil and biofuels attracted the breeders to breed cultivars with increased production [29]. Besides yield improvement, researchers have also been focused on environmental stress and tolerance through engineered cultivars that resist environmental stresses, such as salinity and alkalinity stress [33,34], water stress [35] and nutritional deficiency [36,37,38]. These efforts will improve the final seed yield. Yield is the most important and complex feature in crop plants that reflects the environmental interaction and governs the developmental processes and growth events prevailing the entire lifecycle of the plant [39].

There are three direct factors for seed yield, including seed weight, seed number and silique number per plant. Other factors that indirectly affect the component traits include biomass, harvest index, plant architecture and adaptation, resistance to the biotic and abiotic constraints [40]. Hence, seed yield can be improved by keeping into consideration the direct component features and the other indirect contributing traits. Previous reports described some morphological and agronomic traits, such as siliques (pods) per plant, seed per silique, silique length, seed weight, plant height and oil contents [40,41,42], as yield accelerating traits. These yield accelerating traits can be used based upon selection criteria for yield improvement in rapeseed. Among the contributing traits, siliques per plant (SP) and seeds per silique contribute to the total number of seeds produced by the plant; thus, they directly control and determine the seed yield [43]. Moreover, these traits are further controlled by other plant traits such as plant height, branches per plant, silique density on the plant and silique traits (silique length, number of siliques, etc.). Analyzing the fundamental genetic mechanism and control of these traits separately will help to understand the dependent traits, hence improving seed yield in *B. napus* [44].

## 3. The Role of Genome-Wide Association Studies (GWAS) in Molecular Plant Breeding

The GWAS technique has been efficiently utilized to integrate novel traits in crops, which ameliorate the statistical correlation between genetic markers and phenotypic traits in the various crop varieties within the natural populations [45,46,47]. GWAS is a well-known technique in the framework of human genetics and possesses many useful aspects to cover and straighten out a variety of positive correlations between complicated diseases, as well as common/useful variants, but, due to the missing heritability, which still comes across as a problematic challenge, millions of molecular markers and the majority of individuals would be prerequisite to identify a wide range of QTLs. Nonetheless, in plant breeding, the missing heritability seems to be less serious due to some genetic variants, which explicitly demonstrates the phenotypic variation [48]. More recently, GWAS has been promisingly involved for many crop varieties [15], such as *Oryza sativa* [21], *Zea mays* [22], *Triticum aestivum* [19], *Hordeum vulgare* [18], *Avena sativa* [16], *Brassica napus* [24], *Glycine max* [20], *Arachis hypogaea* [23] and *Sorghum bicolor* [17], cataloged in Table 1. In plant breeding, GWAS methodologies seem to be more successful, because the previous findings coherently demonstrated the greater extent of phenotypic variations over the human GWAS outcomes [48]. The drawback of GWAS is the “fanciful” fabrication between the desired trait of interest and molecular markers. The previous outcomes demonstrated that the cryptic population is one of the significant determinants of fictitious relations [49,50]. Pritchard et al. [51] implicitly visualized the complete population structure that is based on the Bayesian clustering approach (STRUCTURE). They standardized a K population’s model where the individuals were nominated according to their genotype ratio among different population varieties. They also predicted the allele frequency of the population. Price et al. [52] developed a new strategy through the routine practice of principal component analysis in order to interpret the population structure in a given genetic dataset [50] that significantly governs the statistics of “axes of variation”. Moreover, various methods have been proved, although they have shown limited success [51,52]. Apart from these conventional methodologies adopted for phenotypic records and pedigree analysis, modification at the DNA level seems to contribute sufficient information about the principal population structure [53]. In crop breeding, the information about population structure can perform a vital role in establishing the manipulation of competent germplasm [54]. Thus, due to its wide range of applications, GWAS has the efficacy to be utilized directly in plant breeding programs [55].

### The Role of QTLs and GWAS in Three Yield-Related Traits in Rapeseed

Yield is the most important but one of the complex traits in crops. Because of the ever-growing population, the increase in the food demand has been deemed a global concern and is now becoming the major challenge for the speedy breeding of plant cultivars to generate high yield oilseed rape cultivars with increasing agricultural sustainability and productivity to fulfill the burgeoning demand globally [125,126]. Besides pod number, seeds per silique (SS) and thousand-seed weight (TSW) are the other two important yield-determining components of a single plant, both of which are directly associated with ovule development. According to a recent assessment of rapeseed cultivars, the approximated number of seeds per pod is about ~20, which is thought to be far lower than the germplasm ratio, which exhibited a higher range of ~30 [127,128]; this holds the scientists’ interest in the genetic modification and improvement of rapeseed cultivars by means of increasing the number of seed per silique. The most important factors that control the number of seeds per silique include the number of ovary/ovules and the number of unfertile/fertile ovule, as well as the number of fertile ovules that convert into seeds. In *B. napus*, the number of ovules per ovary is measured through different phases occurring during the ovule development [129]. In contrast, the number of unfertile/fertile ovules depends on various fertilization events, i.e., pollen/pollen tubes interaction, pollen sterility and ovule fertility [130,131]. The most desirable outcomes of the fertilized ovule that develops into a seed are principally governed by the biological process of seed development characterized by physiological and nutritional requirements, as well as many other environmental factors, such as abiotic and biotic stimulus [130,132]. QTL mapping for ovule numbers had been studied in other plants, such as *Glycine max* [133], *Vicia faba* [133] and *Raphanus sativa* [134]. More recently, Yuan and Kessler [135] found a locus associated with *NERDI* in *A. thaliana*, which plays a vital role in the regulation of ovule number in both female and male gametophytes. The above discussion suggests that limited literature is available in regard to the genetic basis for ovule number in crops, especially in oilseed rape. To the best of our knowledge, there is no study conducted for ON in *B. napus* yet.

Additionally, the development of high yielding varieties is a major goal in rapeseed breeding, which is determined by three yield components, i.e., siliques per plant (SP), SS and SW [43]. Previous studies articulately observed a negative interaction between silique-related traits. Furthermore, it was also determined that these traits had a derogatory impact on the breeding event by scrutinizing QTNs and genes, which is accomplished for each desirable trait [136,137]. Over the last ten years, TSW has shown rapid development in the field of molecular marker technology [40]. Recently, ~65 and ~168 QTLs, for SS and TSW, respectively, have been examined in 19 linkage groups [37,40,41,43,136,137,138,139,140,141,142,143,144,145,146,147,148,149].

Li et al. [150] detected 133 QTLs for 12 yield-related traits (including SS and TSW, etc.) in rapeseed, containing 14 QTLs consistently identified across two locations. Most of the QTLs were found on the N2 and N7 linkage positions. Radoev et al. [142] also identified 33 QTLs for seed yield and yield component traits at four locations. These results revealed that 10 QTLs had a significant effect on the target traits. On the other hand, Shi et al. [40] employed an extensive study on two populations, i.e., RC-F2 and TNDH, in ten environments. They found 85 QTLs for seed yield and 785 QTLs for 8 yield-related traits (seed number per silique, thousand seed weight, etc.). Bagheri et al. [151] identified 47 QTLs for 17 traits associated with seed yield and plant architecture, which explained from 6% to 56% of the total variance for the targeted traits. Shi et al. [137] found one major QTL (qSN. A06) for SS, which explained 32.1% of the phenotypic variation. Similarly, Yang et al. [129] also found a major QTL for SS on qSN. A06. Raman et al. [152] identified 2 QTLs on Chr A03 and Chr A07, explaining from 5% to 19% of the phenotypic variation for FT and SY. Zhao et al. [153] detected 18 QTLs for SYs and 208 QTLs for YDTs. Recently, Luo et al. [149] studied 22 traits and found 1904 QTLs; among them, 80 QTLs were associated with yield, while 535 QTLs contributed to SY.

Seed size/weight is also an essential factor in Sys [43], having greater heritability than the other yield component traits (YCTs) [40]. In different populations, 6, 4 and 7 QTLs for seed weight were identified located on N7, N17 and N19 linkage groups, respectively [141]. Shi and his group discovered a major QTL qSW.A07-2 [40], while Fan and his colleagues also identified 2 major QTLs TSWA07a and TSWA7b on the same chromosome, which explained from 27.6% to 37.9% of the variation in said trait [41]. Further, Yang and his group detected 1 major QTL (cqSWA09), which also explained high variation (28.2%) for silique length and seed weight [146]. Li et al. [47] carried out a comprehensive study about seed weight and silique length (SL) and 13 and 9 QTLs were identified, which showed the highest variations of 67% and 54%, respectively. Some QTLs were consistently detected through experiments and the authors suggested that these QTLs were more stable and reliable for future study. Using two different populations, 21 and 20 QTLs were identified for seed weight and silique length. The ranges of phenotypic variations for seed weight (SW) and seed length (SL) were observed to be from 24.4% to 62.9% and from 55.1% to 74.3%, respectively [154].

Based on a 60K SNP array, some studies were conducted on various traits of *B. napus* (flowering time and harvest index) [61,155]. Based on a 60K SNP array, Li et al. [47] identified significant SNPs for erucic acid content (A08 and C03), glucosinolates content (A09, C02, C07 and C09) and seed weight (A07 and A09). These results revealed that the identified significant SNPs were suitable for fine mapping complex traits of *B. napus* [47]. Cai et al. [44] employed GWAS for six yield-related traits using 192 inbred lines of rapeseed. They identified seven and nine associated markers for seed per silique and thousand seed weight. These lines were genotyped using 451 SLM markers and 740 AFLP markers. Schiessl et al. [66] studied seed yield and yield-determining traits using a 60K SNP array and identified 36 loci associated with target traits in *B. napus*. GWA studies have not been extensively adopted in the genetic dissection of ovule number, seed per silique and thousand seed weight in rapeseed [66].

**Figure 1:**
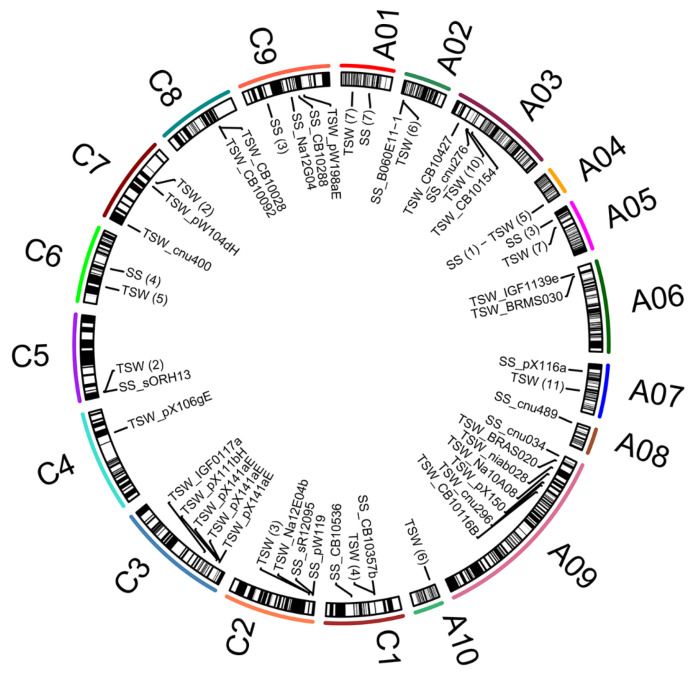
Distribution of QTLs for seeds per silique (SS) and seed weight (TSW) across the chromosomes (A01–A10; C1–C9) of rapeseed. Values between parentheses indicate the number of QTLs for a given trait (for details see Supplementary Table S1).

More recently, Khan et al. [4] carried out a GWAS analysis on 521 accessions of oilseed rape, genotyped with a *Brassica* 60 K SNP array, by using single-locus GWAS (SL-GWAS) and multi-locus GWAS (ML-GWAS) methods. The outcomes of this study presented the significant numbers of 31 and 280 QTLs/QTNs, that were detected by analyzing SL-GWAS and ML-GWAS methodologies, respectively. Among these sequences, 74 common significant QTNs (which include 8 for ovule number (ON), 32 for SS and 34 for TSW) were repeatedly detected by more than three ML-GWAS models and in multiple environments [4]. Figure 1 shows the distribution of important loci associated with seed per silique and thousand-seed weight across the chromosomes of rapeseed by QTL and GWAS studies.

We greatly emphasize that the genetic modification of these traits may also improve rapeseed molecular breeding to develop eco-friendly and improved yield-related cultivars. The outcomes analyzed by this approach may principally favor a strong revolution for the improvement of rapeseed.

## 4. Candidate Genes and Superior Alleles of Seed Yield Related Loci Identified in Rapeseed

The candidate gene approach implies the identification of significant genes that are important for quantitative traits and agriculture. The candidate gene approach was first used for maize (flowering time) [156,157] and then also used for many important traits [158,159]. Recently, in *B. napus* three independent pieces of literature were available on candidate genes related to YDTs [126]. Zhao and his group identified candidate genes for seed yield (4), TSW (2) and plant height (1) [33]. Zheng and his colleagues also detected candidate genes, 31 for plant height, 15 for branch initiation height and 17 for branch number using diverse oilseed rape accessions [69]. Lu et al. [82] used 520 accessions of rapeseed and discovered 6, 7, 7 and 3 candidate genes for seed per silique, pod number, branch pod number and TSW, respectively. Khan et al. [4] further prophesied the genes associated with SS, TSW and ON, respectively. They found a total of 42 candidate genes, which were homologous to *A. thaliana* yield-determining traits, which lie in the range of QTLs. The candidate genes for ON, SS and TSW were identified in the numbers of 3, 17 and 20, respectively, whereas 2 candidate genes were linked with SS/TSW (*Bn-A09-p30391674/Bn-A09-p30404228*) or TSW/SS (*Bn-A08-p16523108/Bn-scaff_16665-p54637*).

**Figure 2:**
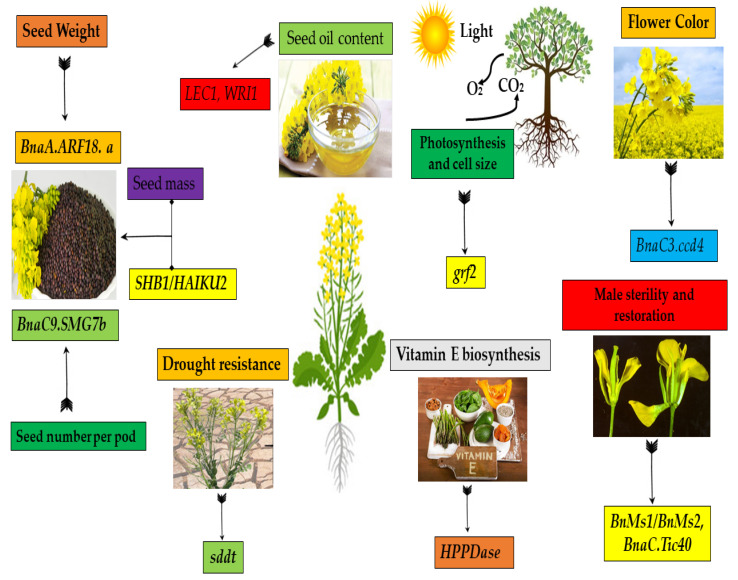
This figure presents the major role of cloned genes for key traits in rapeseed, viz: yield, drought stress resistance, seed oil, flower color, disease resistance, photosynthetic rate, etc.

In rapeseed, only two findings were reported that demonstrated the gene cloning for TSW and SS, respectively. For SS, *qSS.C9*, the QTL is thought to be one of the important QTLs [160] that play a significant role in developing female gametophytes [131]. Additionally, the *ARF18* gene controls both TSW and SL QTL formation [161]. Moreover, the *ARF18* gene slowed down the function of the auxin gene and had an inhibitory effect on its biological activity, which was assumed to modulate the activity of silique wall development and TSW by regulating maternal genes [161]. Furthermore, many other genes also play a tremendous role in cloning many other important traits, viz. yield, abiotic stress, seed oil, disease resistance, etc., as presented in Figure 2.

More recently, Ma et al. [13] identified, in maize crop, 63 common QTNs within the 31 elite inbred lines. Of them, 36 QTNs were showed in <50% superior alleles, which anticipated that these identified alleles were not appropriate for the artificial selection method, whereas 27 further QTNs were identified to be >50%; therefore, these findings confirmed that these alleles were the best match for artificial selection [13]. Subsequently, the recent outcomes of Khan et al.’s study [4] identified 74 significant QTNs by utilizing multiple methods in a different environment. The identified QTNs were determined to be significant QTNs that were strongly associated with yield-determining traits. Of these, 8, 13 and 34 QTNs were observed to have superior alleles for ON, SS and TSW, respectively. In fact, the allele percentage ranged from 9.4% to 85.41%, from 7.67% to 82.34% and from 4.79% to 68.13%, for SS, TSW and ON, respectively. Interestingly, one of the identified QTN for ON (*Bn-A09-p10297982*) had the highest percentage proportion (67%), whereas the remaining seven showed less than 50%. For SS, 31 QTNs were identified, in which 13 had superior alleles >50%, whereas 18 of the identified QTNs had <50% allele ratio. Lastly, for TSW, among 34 identified QTNs, 14 QTNs showed superior alleles for more than 50%, whereas 20 QTNs showed fewer superior alleles, about 50%. Remarkably, the single QTNs for both TSW (*Bn-A03-p24823015*) and SS (*Bn-A03-p403559*) had the highest proportion of superior alleles, 82.34% and 85.41%, respectively. Therefore, the authors argued that these identified superior alleles showed an effective role in the above-aforementioned yield-determining traits. We strongly believe that the obtained high percentage of superior alleles will be convenient for high yield production in the revolution of rapeseed breeding. Until now, there is no significant study organized to find the superior alleles in rapeseed. In the marker-assisted selection (MAS) method, the highest percentage of superior alleles is very accessible. This strategy also showed significant insights into seed yield and other useful economic traits in many plant species.

## 5. Comparative Overview of Single-Locus and Multi-Locus GWAS Methodologies

To improve yield-determining traits (YDTs), contributing to a better understanding of their genetic basis and diversity, recently, genome-wide association studies (GWAS) approaches have been extensively used to dissect the complex traits in crops. Before this, most of the findings have been reported to have utilized single-locus GWAS, such as the mixed linear model (MLM) ([162,163]), while, recently, various new MLM-based models have been introduced [164]. More comprehensively, these novel strategies have various applications in the genetic integration of novel and omics-related traits, facilitating the recent breakthrough in the generation of bioinformatics and sequencing strategies. Additionally, single-locus models have some pitfalls, including the GLM principally showing high false-positive rates (FPR). In contrast, MLM used Bonferroni corrections for the identification of loci to mitigate the chances of FPR, though this process is very rigorous and the outcomes for some important loci data are still missing. To overcome these limitations, Zhang et al. [165] utilized different multi-locus GWAS approaches, including multi-locus random-SNP-effect mixed linear model (mrMLM) [166], iterative Modified-Sure Independence Screening EM-Bayesian LASSO (ISIS EM-BLASSO) [167], fast multi-locus random-SNP-effect EMMA (FASTmrEMMA) [168] and pLARmEB [169]. In a previous study, Li et al. [170] demonstrated that the comprehensive approach of GWAS is very effective in identifying the number of QTNs, more specifically in *B. napus*, by utilizing the ML-GWAS and SL-GWAS methods.

The mrMLM method enhances the identification of loci by more than 55% throughout the covered region. For instance, Misra et al. [171] determined the significant rice variants in determining rice grain by employing ML-GWS and SL-GWAS methods. Therefore, the combined utilization of the ML-GWAS and SL-GWAS methodologies was useful to detect the genetic locus of *GWi7.1, GWi7.2* and to identify novel genes. Moreover, Xu et al. [172] utilized multi-locus and single-locus GWAS methods to quantify the significance of novel QTNs in the pasting traits of maize starch. In contrast to the ML and SL-GWAS methods, it was confirmed that ML-GWAS FASTmrEMMA had novel QTNs (29), whereas SL-GWAS (GEMMA) showed a much lower number of novel QTNs (7) [172]. More recently, Peng et al. [121] utilized six ML-GWAS methods to determine the genetic dissection of 20 amino acid levels in *Triticum aestivum* L. As a result, they achieved that ML-GWAS models are very authentic and dynamic [121]. Correspondingly, Cui et al. [112] confirmed that, via this multi-locus GWAS approach, most of the QTNs were discovered by following ISIS EM-BLASSO [112]. Additionally, Su et al. [173] showed research findings in the genetic dissection of upland cotton, in which 70 QTNs were identified. They concluded that the ML-GWAS methods are much more powerful and authentic than the single-locus GWAS (MLM) methods in TASSEL v5.0 [173]. Finally, the results mentioned above confirm the strengths of ML-GWAS strategies as compared to SL-GWAS methods, although, more recently, the outcomes of some studies recommend that the co-interaction of single-locus and multi-locus GWAS methods significantly enhances the identification of rationality and robustness of GWAS [97,115,165,172].

## 6. Conclusions, Future Trends, and Perspectives

This review presented the robustness and power of QTL/GWAS analyses, candidate gene association studies and superior allele identification in *Brassica napus*. Follow-up work can be carried out on the following aspects:

1. The genetic analysis of phenotypic traits has found novel and significant QTNs/QTLs/candidate genes, giving research a chance to progress, more specifically, in the identification of the activity of these genes and further recognize the mechanism of the genetic constitution of the traits of thousand seed weight, ovule number per ovary and seed number per silique through crosswise adverse geographical and climatic conditions.
2. The information collected from previous studies enriches the knowledge of variations in populations in the three above-mentioned yield-related traits; therefore, this warrants the need for supplementary authentication that will deliver markers through the integration into the host plant improvement.
3. The association analysis of the candidate gene method and the development of near-isogenic, transgenic, or mutant plants may be efficiently utilized to identify novel and significant alleles and how they interact with the above-mentioned yield-related traits.
4. Genomic regions reviewed in the present study merit attention for their further utilization in breeding programs that use marker-assisted selection (or genomics-assisted breeding) on *Brassica napus*.

## Figures and Tables

**Table 1:** The enigmatic role of genome-wide association studies in various crop species.

Crop	Population Size	Markers	Traits	References
*Aegilops tauschii*	322	7185 SNPs	Morphological traits	[56]
*B. napus*	523	41 SNPs	Flowering time	[57]
*B. napus*	248	60K SNPs	Seed germination and vigor	[58]
*B. napus*	472	60,000 SNPs	Seed weight and quality	[47]
*B. napus*	155	35,791 SNPs	Harvest index	[59]
*B. napus*	521	60K SNPs	Seed oil content	[60]
*B. napus*	523	60K SNPs	Flowering time	[61]
*B. napus*	348	60K SNPs	Silique related traits	[62]
*B. napus*	472	19,945 SNPs	Plant height and primary branch number	[63]
*B. napus*	143	60K SNPs	Branch angle	[64]
*B. napus*	520	60K SNPs	Branch angle trait	[65]
*B. napus*	158	60K SNPs	Flowering time and yield responses	[66]
*B. napus*	192	369 SSR, 740 AFLP	Yield-related traits	[44]
*B. napus*	370	60K SNPs	Quantity of fatty acids	[67]
*B. napus*	422	60K SNPs	Root development	[68]
*B. napus*	333	60K SNPs	PH, BIH, and BN	[69]
*B. napus*	588	385,692 SNPs	Oil content	[70]
*B. napus*	300	201,187 SNP	Fatty acid composition	[71]
*B. napus*	216	30,262 SNPs	Root architectural traits	[72]
*B. napus*	157	742 SNPs	Seed weight and silique length	[73]
*B. napus*	327	33,186 SNPs	Branching morphogenesis.	[74]
*B. napus*	435	60K SNPs	Fatty acid composition and content	[75]
*B. napus*	331	60K SNPs	Silique number	[76]
*B. napus*	307	60K SNPs	Seed glucosinolates contents	[77]
*B. napus*	419	60K SNPs	Seedling stage	[78]
*B. napus*	300	201,817 SNPs	Earliness traits	[79]
*B. napus*	368	60K SNPs	Salt tolerance-related traits	[34]
*B. napus*	520	60K SNPs	Fatty acid composition	[80]
*B. napus*	210	23,435 SNPs	Hypocotyl elongation	[81]
*B. napus*	520	*Brassica* 60K	Seven yield-determining traits	[82]
Barley	175	107 SSRs	Seed aging and longevity	[83]
Barley	~500	1536 SNPs	Morphological trait	[84]
Barley	122 DH lines	9680 SNPs	14 main agronomic traits	[85]
Barley	275	9K SNP, 3072 SNP	Six-rowed spring barley	[86]
Barley	233	7864 SNPs	Root and shoot architecture traits	[87]
Barley	25	9 K iSelect SNPs	Plant growth under drought stress	[88]
Barley	222-2-303 6-rowed	7864 SNPs.	Adherence of hulls to the caryopsis	[89]
Barley	109	15,828 DArTseqs and 7829 SNPs	Chlorophyll fluorescence induction (OJIP) parameters	[90]
Barley	166	777 DArT markers	Drought stress	[91]
Cotton	169	53,848 SNPs	Fiber quality traits	[15]
Cotton	319	55,060 SNPs	Drought stress	[92]
Cotton	231	122 SSR and 4729 SNP markers	Fiber quality traits and yield components	[93]
Cotton	196	CottonSNP80K	Salt tolerance	[94]
Cotton	316	81,675 SNP	Drought tolerance	[95]
Cotton	83	15,369 SNPs	Oil content	[96]
Cotton	215	~1.57 million SNPs	Salt tolerance	[97]
Flax	370	258,873 SNPs	Pasmo resistance	[98]
Foxtail millet	916	0.8 m SNPs	Agronomic traits	[99]
Maize	384	681,257 SNPs	Seedling root architecture traits	[100]
Maize	999	56,110 SNPs	Northern corn leaf blight resistance	[101]
Maize	368	1.03 m SNPs	Kernel oil concentration fatty acid composition	[102]
Maize	25000	SNPs	Southern leaf blight resistance	[103]
Maize	-	SNPs	Leaf architecture	[104]
Maize	144	43,427 SNPs	The regenerative capacity of the embryonic callus	[13]
Maize	257	48,193 SNPs	Stalk lodging resistance	[14]
Maize	230	145,232 SNPs	Starch pasting properties	[61]
Rice	413	44,100 SNPs	Agronomic traits	[105]
Rice	9	71,710 SNPs	Agronomic traits	[106]
Rice	220	6000 SNPs	Salinity tolerance	[107]
Rice	517	∼3.6 m SNPs	Agronomic traits	[108]
Rice	950	SNPs	Flowering time grain-related traits	[109]
Rice	20	32,655 SNPs	Agronomic traits	[110]
Rice	236	147,692 SNPs	Cooked rice texture	[111]
Rice	478	162,529 SNPs	Salt-tolerance	[112]
Sorghum	971	~26,500 SNPs	Plant height and architecture	[17]
Sorghum	245	85,885	Forage quality-related traits	[113]
Soybean	96	SSRs	Plant height pods/plant 100-seed weight plant growth habit seeds/pod days to 50% flowering and maturity	[114]
Soybean	368	62,423 SNPs	Plant height and the number of nodes	[115]
Soybean	219	292,035 SNPs	Photosynthetic response to low P stress	[116]
Soybean	144	SoySNP660k	Protein content	[117]
Sugarcane	20	20 SSRs	Cane weight tillers/plant	[118]
Tomato	174	182 SSRs	Flavor traits	[119]
Wheat	382	SNPs	Agronomic traits and carbon isotope	[120]
Wheat	182	14,646 SNPs	20 free amino acid	[121]
Wheat	339	13,098 SNPs	Karnal bunt resistance	[122]
Wheat	160	10,172 SNPs	Wheat quality and yield-related traits	[123]
Wheat	635	10,802 SNPs	Flour yield and alveograph quality traits	[124]

## Data Availability

Data are contained within the article.

## Supplementary Materials

The following are available online at https://www.mdpi.com/article/10.3390/biom11101516/s1, Table S1: Summary of the identified loci associated with seed per silique (SS) and thousand-seed weight (TSW) in rapeseed by QTL and GWAS studies.
