# Supplementary material for: Advances and Challenges for QTL Analysis and GWAS in the Plant-Breeding of High-Yielding: A Focus on Rapeseed
Source: Biomolecules. 2021 Oct 15;11(10):1516. doi: 10.3390/biom11101516 (PMC8533950; doi:10.3390/biom11101516)
Supplement: Supplementary file 1 [file biomolecules-11-01516-s001.zip › biomolecules-1370748-supplementary.pdf]

Table S1. Summary of the identified loci associated with seed per silique (SS) and thousand-seed weight (TSW) in rapeseed by QTL and GWAS studies.

Trait	Marker name	Chromosome	Marker position (Mb)*		Reference
			Start (Mb)	End(Mb)	
**SS**	Na14-G06	A01	8.24	9.24	[1]
**SS**	cnu034	A09	1.2	2.2	[1]
**SS**	MD21	A05	0.81	1.81	[2]
**SS**	CB10357b	C01	17.36	18.36	[2]
**SS**	CB10536	C01	35.27	36.27	[2]
**SS**	SCC9005	C09	44.59	45.59	[3]
**SS**	SRC9022	C09	46.03	47.03	[3]
**SS**	BnGMS464	A01	21.03	22.03	[4]
**SS**	B060E11-1	A02	2.15	3.15	[4]
**SS**	SA79	A05	3.61	4.61	[4]
**SS**	FITO035	C06	28.64	29.64	[4]
**SS**	CB10632	C06	33.75	34.75	[4]
**SS**	niab71	A01	1.32	2.32	[5]
**SS**	cnu142	A01	1.45	2.45	[5]
**SS**	niab096	A01	1.99	2.99	[6]
**SS**	IGF9014b	A01	6.31	7.31	[5]
**SS**	IGF2071e	A01	17.54	18.54	[5]
**SS**	cnu276	A03	15.23	16.23	[6]
**SS**	cnu256	A04	16.68	17.68	[6]
**SS**	BRAS063	A05	3.32	4.32	[6]
**SS**	pX116a	A07	5.22	6.22	[6]
**SS**	cnu489	A08	0.28	1.28	[6]
**SS**	pW119	C02	1.44	2.44	[6]
**SS**	sR12095	C02	1.74	2.74	[5]
**SS**	sORH13	C05	2.25	3.25	[7]
**SS**	CB10010	C06	20.48	21.48	[5]
**SS**	AP1a	C06	27.15	28.15	[6]
**SS**	pW233b	C09	14.44	15.44	[5]
**SS**	Na12G04	C09	33.51	34.51	[5]
**SS**	CB10288	C09	39.39	40.39	[6]
**TSW**	Ra3-H10	A05	19.45	20.45	[8]
**TSW**	cnu296	A09	27.13	28.13	[8]
**TSW**	CB10597	A01	2.5	3.5	[2]
**TSW**	MR12	A03	17.65	18.65	[2]
**TSW**	CB10154	A03	20.24	21.24	[9]
**TSW**	MD21	A05	0.81	1.81	[2]

**TSW**	MR119	A05	5.74	6.74	[2]
**TSW**	Na12B02	A07	5.02	6.02	[9]
**TSW**	Na12E11	A07	11.78	12.78	[9]
**TSW**	BRAS020	A09	5.37	6.37	[9]
**TSW**	Ol10B11	A10	0.41	1.41	[9]
**TSW**	Na12E09	A10	4.78	5.78	[9]
**TSW**	CB10357b	C01	17.36	18.36	[2]
**TSW**	CB10536	C01	35.27	36.27	[2]
**TSW**	Na12E04b	C02	4.47	5.47	[2]
**TSW**	CB10234	C06	13.14	14.14	[2]
**TSW**	Ol10F08	C06	20.03	21.03	[9]
**TSW**	CB10597	A01	2.5	3.5	[9]
**TSW**	CB10116B	A09	33.35	34.35	[9]
**TSW**	CB10357b	C01	17.36	18.36	[9]
**TSW**	Na12E04b	C02	4.47	5.47	[9]
**TSW**	BnGMS103	A02	11.11	12.11	[10]
**TSW**	CB10471b	A02	15.86	16.86	[10]
**TSW**	sR0282R	A07	1.64	2.64	[11]
**TSW**	B060E11-1	A02	2.15	3.15	[12]
**TSW**	B070J05	A02	2.41	3.41	[12]
**TSW**	CB10540	A02	22.11	23.11	[12]
**TSW**	cnu252	A04	15.38	16.38	[12]
**TSW**	BnGMS608	A07	19.79	20.79	[12]
**TSW**	niab028	A09	11.43	12.43	[12]
**TSW**	MR156	A10	0.5	1.5	[12]
**TSW**	Na12E09	A10	4.78	5.78	[12]
**TSW**	FITO043	C06	28.64	29.64	[12]
**TSW**	CB10028	C08	36.35	37.35	[12]
**TSW**	pX136bE	C07	2.53	3.53	[13]
**TSW**	pW186cH	A07	0.86	1.86	[14]
**TSW**	pX126fH	A07	0.88	1.88	[13]
**TSW**	pW162eE	A07	7.85	8.85	[14]
**TSW**	pX111bH	C03	9.25	10.25	[14]
**TSW**	pX106gE	C04	48.59	49.59	[14]
**TSW**	pW104dH	C07	36.13	37.13	[14]
**TSW**	pW198aE	C09	43.33	44.33	[13]
**TSW**	CB10597	A01	2.5	3.5	[15]
**TSW**	sN3523R	A01	18.77	19.77	[15]
**TSW**	CB10095	A01	6.81	7.81	[6]
**TSW**	BRAS078	A01	8.06	9.06	[5]
**TSW**	IGF2071e	A01	17.54	18.54	[5]

**TSW**	FITO40	A02	1.61	2.61	[6]
**TSW**	pX141eE	A03	4.85	5.85	[5]
**TSW**	pX141eE	A03	4.99	5.99	[6]
**TSW**	pX141eE	A03	5.01	6.01	[5]
**TSW**	CB10427	A03	7.49	8.49	[6]
**TSW**	IGF3125a	A03	14.29	15.29	[5]
**TSW**	Ol11G11a	A03	14.78	15.78	[6]
**TSW**	cnu270	A03	15.86	16.86	[6]
**TSW**	cnu002	A03	16.14	17.14	[5]
**TSW**	cnu215	A03	17.92	18.92	[6]
**TSW**	cnu371	A03	20.77	21.77	[6]
**TSW**	pX129b	A04	4.25	5.25	[6]
**TSW**	sN13034	A04	7.86	8.86	[6]
**TSW**	sN2025	A04	11.52	12.52	[6]
**TSW**	cnu256	A04	16.68	17.68	[5]
**TSW**	sN12353a	A05	4.4	5.4	[6]
**TSW**	IGF0149c	A05	8.02	9.02	[6]
**TSW**	cnu325	A05	9.25	10.25	[6]
**TSW**	pW247	A05	20.48	21.48	[5]
**TSW**	IGF1139e	A06	3.31	4.31	[5]
**TSW**	BRMS030	A06	5.12	6.12	[6]
**TSW**	cnu063	A07	0.76	1.76	[5]
**TSW**	sR0282R	A07	1.64	2.64	[5]
**TSW**	IGF2021z	A07	4.71	5.71	[5]
**TSW**	BRAS023	A07	10.11	11.11	[6]
**TSW**	niab90	A08	12.18	13.18	[5]
**TSW**	IGF1108c	A08	14.1	15.1	[6]
**TSW**	Na10A08	A09	17.18	18.18	[6]
**TSW**	pX150	A09	24.48	25.48	[6]
**TSW**	sN8502	A10	1.94	2.94	[7]
**TSW**	Na10D07	A10	10.96	11.96	[7]
**TSW**	Na12C08	C01	21.93	22.93	[6]
**TSW**	pW119	C02	1.44	2.44	[6]
**TSW**	sR12095	C02	1.74	2.74	[5]
**TSW**	pX141aE	C03	6.52	7.52	[6]
**TSW**	pX141aE	C03	6.68	7.68	[6]
**TSW**	pX141aE	C03	6.7	7.7	[6]
**TSW**	IGF0117a	C03	20.46	21.46	[5]
**TSW**	sORH13	C05	2.25	3.25	[7]
**TSW**	Ol10B02	C05	2.78	3.78	[5]
**TSW**	sR12387	C06	29.49	30.49	[6]

**TSW**	pW134	C06	29.58	30.58	[6]
**TSW**	cnu400	C07	1.63	2.63	[5]
**TSW**	pW179b	C07	40	41	[5]
**TSW**	CB10092	C08	36.04	37.04	[7]

*Based on Raboanatahiry et al. [16].

References

1. Qi, L.; Mao, L.; Sun, C.; Pu, Y.; Fu, T.; Ma, C.; Shen, J.; Tu, J.; Yi, B.; Wen, J. Interpreting the genetic basis of silique traits in *Brassica napus* using a joint QTL network. *Plant Breed.* **2014**, *133*, 52–60.
2. Radoev, M.; Becker, H.C.; Ecke, W. Genetic analysis of heterosis for yield and yield components in rapeseed (*Brassica napus* L.) by quantitative trait locus mapping. *Genetics* **2008**, *179*, 1547–1558.
3. Zhang, L.; Li, S.; Chen, L.; Yang, G.J.T.; Genetics, A. Identification and mapping of a major dominant quantitative trait locus controlling seeds per silique as a single Mendelian factor in *Brassica napus* L. *Theor. Appl. Genet.* **2012**, 125, 695–705.
4. Wang, X.; Chen, L.; Wang, A.; Wang, H.; Tian, J.; Zhao, X.; Chao, H.; Zhao, Y.; Zhao, W.; Xiang, J. Quantitative trait loci analysis and genome-wide comparison for silique related traits in *Brassica napus*. *BMC Plant Biol.* **2016**, 16, 71.
5. Shi, J.; Li, R.; Zou, J.; Long, Y.; Meng, J. A dynamic and complex network regulates the heterosis of yield-correlated traits in rapeseed (*Brassica napus* L.). *PLoS ONE* **2011**, 6, e21645.
6. Shi, J.; Li, R.; Qiu, D.; Jiang, C.; Long, Y.; Morgan, C.; Bancroft, I.; Zhao, J.; Meng, J. Unraveling the complex trait of crop yield with quantitative trait loci mapping in *Brassica napus*. *Genetics* **2009**, 182, 851–861.
7. Luo, Z.; Wang, M.; Long, Y.; Huang, Y.; Shi, L.; Zhang, C.; Liu, X.; Fitt, B.D.; Xiang, J.; Mason, A.S.; et al. Incorporating pleiotropic quantitative trait loci in dissection of complex traits: Seed yield in rapeseed as an example. *Theor. Appl. Genet.* **2017**, 130, 1569–1585.
8. Fu, Y.; Wei, D.; Dong, H.; He, Y.; Cui, Y.; Mei, J.; Wan, H.; Li, J.; Snowdon, R.; Friedt, W. Comparative quantitative trait loci for silique length and seed weight in *Brassica napus. Sci. Rep*. **2015**, 5, 1–9.
9. Basunanda, P.; Radoev, M.; Ecke, W.; Friedt, W.; Becker, H.; Snowdon, R. Comparative mapping of quantitative trait loci involved in heterosis for seedling and yield traits in oilseed rape (*Brassica napus* L.). *Theor. Appl. Genet.* **2010**, 120, 271.
10. Zhang, L.; Yang, G.; Liu, P.; Hong, D.; Li, S.; He, Q. Genetic and correlation analysis of silique-traits in *Brassica napus* L. by quantitative trait locus mapping. *Theor. Appl. Genet.* **2011**, 122, 21–31.
11. Fan, C.; Cai, G.; Qin, J.; Li, Q.; Yang, M.; Wu, J.; Fu, T.; Liu, K.; Zhou, Y. Mapping of quantitative trait loci and development of allele-specific markers for seed weight in *Brassica napus. Theor. Appl. Genet.* **2010**, 121, 1289–1301.
12. Zhao, W.; Wang, X.; Wang, H.; Tian, J.; Li, B.; Chen, L.; Chao, H.; Long, Y.; Xiang, J.; Gan, J. Genome-wide identification of QTL for seed yield and yield-related traits and construction of a high-density consensus map for QTL comparison in *Brassica napus. Front. Plant Sci.* **2016**, 7, 17.
13. Quijada, P.A.; Udall, J.A.; Lambert, B.; Osborn, T.C. Quantitative trait analysis of seed yield and other complex traits in hybrid spring rapeseed (*Brassica napus* L.): 1. Identification of genomic regions from winter germplasm. *Theor. Appl. Genet.* **2006**, 113, 549–561.
14. Udall, J.A.; Quijada, P.A.; Lambert, B.; Osborn, T.C. Quantitative trait analysis of seed yield and other complex traits in hybrid spring rapeseed (*Brassica napus* L.): 2. Identification of alleles from unadapted germplasm. *Theor. Appl. Genet.* **2006**, 113, 597–609.
15. Yang, P.; Shu, C.; Chen, L.; Xu, J.; Wu, J.; Liu, K. Identification of a major QTL for silique length and seed weight in oilseed rape (*Brassica napus* L.). *Theor. Appl. Genet.* **2012**, 125, 285–296.
16. Raboanatahiry, N.; Chao, H.; Dalin, H.; Pu, S.; Yan, W.; Yu, L.; Wang, B.; Li, M. QTL alignment for seed yield and yield related traits in *Brassica napus*. *Front. Plant Sci.* **2018**, *9*, 1127.
